# 3D image superimpositions for orthodontic diagnostics and therapy

**DOI:** 10.1186/1746-160X-10-S1-O7

**Published:** 2014-12-12

**Authors:** Christos Katsaros

**Affiliations:** 1 Department of Orthodontics and Dentofacial Orthopedics, University Hospital Bern, Berne, Switzerland

## 

Orthodontists and maxillofacial surgeons have always been interested to quantify the influence of orthodontic and/or orthognathic surgery treatment on craniofacial morphology.

Historically, superimpositions of cephalometric radiographs have been used to study growth and treatment effects. In recent years advances in 3D imaging techniques have led to their widespread use for setting treatment goals, choosing treatment modalities, predicting treatment results and evaluating treatment and/or growth changes.

Various different techniques have been reported for superimposition of 3D datasets derived from either conventional computed tomography (CT) or lower radiation cone beam CT (CBCT) images.

This lecture presents the main 3D superimposition techniques that are currently available and discuss their unique specifications and inherent limitations.

